# Demographic differences in public acceptance of waste-to-energy incinerators in China: High perceived stress group vs. low perceived stress group

**DOI:** 10.3389/fpsyg.2022.948653

**Published:** 2022-10-26

**Authors:** Jiabin Chen, Xinyao He, Ye Shen, Yiwei Zhao, Caiyun Cui, Yong Liu

**Affiliations:** ^1^School of Civil Engineering and Architecture, Zhejiang Sci-Tech University, Hangzhou, China; ^2^School of Civil Engineering and Architecture, North China Institute of Science and Technology, Langfang, China

**Keywords:** waste-to-energy incineration project, public acceptance, demographic characteristic, perceived stress, China

## Abstract

Demographic characteristics have been recognized as an important factor affecting public acceptance of waste-to-energy (WTE) incineration facilities. The present study explores whether the differences in public acceptance of WTE incineration facilities caused by demographic characteristics are consistent in residential groups under different perceived stress using data collected by a large-scale questionnaire survey (1,066 samples) conducted in three second-tier cities in China. The result of data analysis using a T-test (one-way ANOVA) shows firstly that people with low perceived stress have higher public acceptance of WTE incineration facilities. Second, the differences in public acceptance of WTE incineration facilities caused by demographic characteristics (gender, educational attainment, and age) vary in residential groups with different perceived stress levels. The findings enrich the knowledge system related to demographic characteristics research on NIMBY infrastructure projects and provide the theoretical basis for the government to formulate more targeted policies about NIMBY infrastructure sitting.

## Introduction

The global annual output of municipal solid waste has reached 1.3 billion tons, and it is expected to reach 2.2 billion tons/year in 2025, with an increase of 70% (Fang and Li, 2019). With the rapid increase of municipal solid waste, more and more attention has been paid to the issue of how to treat municipal solid waste more efficiently and environmentally (Kalyani and Pandey, 2014). Compared with the current mainstream landfill treatment, WTE incineration provides a more reasonable solution for the field of municipal solid waste disposal. Specifically, WTE incineration can effectively reduce the volume of solid waste, thus protecting land resources and realizing sustainable development of clean energy utilization (Dudley, 2018). Therefore, whether from the point of view of ecological environment protection or energy utilization, WTE incineration is considered to be the best option for solid waste treatment (Ogunjuyigbe et al., 2017).

However, with the popularization of WTE incineration facilities, a “double paradox” appears in the field of garbage disposal. Although the public knows its important role in the process of sustainable waste treatment, the negative impact of WTE incineration facilities makes the residents near the facilities very excluded (Zhang et al., 2015). This social phenomenon that residents oppose the development of “potentially hazardous facilities” in the local area is also called the NIMBY (Not In My Backyard) effect (Schively, 2007). NIMBY conflicts have not only caused great trouble to the government in urban safety management but also caused great economic losses (Song et al., 2017). Therefore, the siting of WTE incineration facilities is not only a technical challenge but also a complex combination of social, economic, environmental, and technical problems (Mah et al., 2014).

Previous studies have focused on the public acceptance of WTE incineration projects to measure the public response to the facilities (Gunter and Harris, 1998). As an important individual feature, demographic characteristics are considered to be closely related to public acceptance (Flynn et al., 1994). However, research on this issue is currently insufficient. First, although demographic characteristics such as gender, educational attainment, age, race, income level have been proved to affect public acceptance directly (Kahan et al., 2007; Wang and Kim, 2019), some studies have shown that the demographic characteristics of residents living in high-stress communities near potentially hazardous facilities have no significant impact on public acceptance (Yang et al., 2006; Weiner et al., 2013). Second, previous research on the influence of demographic characteristics on public acceptance mainly has focused on residents living in high-stress communities near potentially hazardous facilities (Gustafsod, 1998), with research targeting laypeople (those living further away), an important public opinion base group in the process of building potentially hazardous facilities, mostly lacking. Third, whether the difference of public acceptance of WTE incineration facilities caused by demographic characteristics is consistent in residential groups under different perceived stress has not been fully demonstrated.

To fill the above knowledge gap, the current study attempts to explore the relationship between demographic characteristics and public acceptance in different perceived stress groups using data collected by a large-scale questionnaire survey (1,066 samples) conducted in three second-tier cities in China. The findings enrich the knowledge system related to demographic characteristics research on public acceptance and provide the theoretical basis for the government to formulate more targeted policies about NIMBY infrastructure sitting.

## Literature review

### Public acceptance and demographic characteristics

Public acceptance is often used to measure the public’s general attitude towards new technologies and express their willingness to accept new technologies (Sun and Zhu, 2014). Du and Zhu (2019) divide the factors influencing public acceptance of potentially hazardous facilities into two categories: personal factors and institutional environment factors. On the one hand, institutional and environmental factors, such as public participation and information disclosure, have been proved to have a direct impact on public acceptance (He et al., 2013; Wu, 2017). On the other hand, individual factors such as demographic factors (gender, age, educational attainment, etc.) and social-psychological state (perceived risks, perceived fairness, perceived benefits, etc.) have also been verified in previous studies (Kikuchi and Gerardo, 2009; Besley, 2010; Roh and Kim, 2017).

To carry out this research better, this paper summarizes the influence of demographic characteristics on public acceptance of potentially hazardous facilities. Demographic characteristics that affect public acceptance include gender, age, race, educational attainment, income level, distance from potentially hazardous facilities, and so on. A substantial body of studies showed that white, well-educated, high-income, males and people living far away from facilities have higher public acceptance (Wang and Kim, 2019). First, white male is considered to have higher rights, status, and social trust, so their attitudes and views on new technologies and potentially hazardous facilities are different from others (Kahan et al., 2007). The typical “white male” effect holds that white males not only have much less perceived risks than others but also have greater perceived benefits than others, which makes them more likely to accept potentially hazardous facilities (Flynn et al., 1994). In another survey across the United States, Britain, and Switzerland, it was concluded that the different attitudes of gender towards nuclear energy were interpreted as different roles of gender in risk perception: females pay more attention to nuclear energy and worry about its negative benefits, while male mostly regards this issue as a scientific and technological issue (Rabow et al., 1990). Secondly, the number of people who support the construction of potentially hazardous facilities increases with age, because with the increased age, the elder is more aware of the help of facilities to the local economy (Greenberg and Truelove, 2011). Moreover, the elderly realizes the importance of more energy regeneration and new energy transition, so they have become staunch supporters of the construction of nuclear and renewable energy facilities (Greenberg, 2009). Thirdly, scholars from different countries have proved that when respondents have better educational backgrounds, they are more likely to have a clear understanding of the benefits of potentially hazardous facilities and are more willing to accept potentially hazardous facilities (Choi et al., 2000; Stoutenborough et al., 2013; Sun and Zhu, 2014). Finally, in the study of the distance of hazardous facilities in the residential area, the residents living near the nuclear power plant and ordinary people have completely different views on nuclear power plant facilities, because the closer they live, the more intuitively they can feel the risks brought by the potentially hazardous facilities, and the more they are excluded from building such facilities near their residential areas, which is a typical “NIMBY “effect (Van der Horst, 2007).

### Perceived stress

Lazarus (1966), a well-known psychologist, put forward the concept of psychological stress. Psychological stress refers to a subjective psychological feeling produced by individuals when they realize that the requirements of the internal and external environment threaten them or exceed their coping ability. The study of stressors usually includes two aspects. On the one hand, Holmes and Rahe (1967) concluded the source of individual’s psychological stress as the major events in life, which lead to the loss of internal balance of the body and prompt individuals to make new self-adjustments. On the other hand, Lazarus and Folkman (1984) summarized that the source of psychological stress is not major life events but small annoyances in daily life. The constant accumulation of these annoyances consumes people’s energy and physical strength and ultimately harms their health.

Stress is closely related to the physical and mental health of individuals (Lupien et al., 2009). Moderate stress can improve personal health and the ability to adapt to social life. However, too strong and persistent stress hurts physical and mental health, affecting people’s perception and social behavior (Pengilly and Dowd, 2000). According to the theory of self-defense mechanism, when faced with stress, the human body’s coping is an unconscious psychological defense mechanism, and individuals will unconsciously use defense mechanisms such as denial, evasion, and projection to deal with problems (Freud, 2018). Therefore, there are significant differences between individual perception and social behavior in stress groups and non-stress groups, which have also been verified by many experiments.

First, perceived stress enhances the perceived risk of individuals. The results of an experiment on stress and perceived risk of rescue workers show that rescue workers face the risk of injury repeatedly during work, and this psychological stress makes the risk perception of rescue workers higher than that of ordinary people (Schneider and Bengel, 2014). Besides, the same conclusion is also reflected in the experiment on risk-taking. Nowacki et al. (2019) indicated that compared with people without stress, people with psychosocial stress are less willing to take risks when they face new decisions. Second, high perceived stress reduces one’s trust in others. Potts et al. (2019) pointed out in an experiment to explore the relationship between acute stress and prosocial decision-making that acute stress reduces the tendency of personal trust and changes people’s decision-making behavior. Finally, the high perceived stress affects residents’ perceived fairness. This conclusion was confirmed in an experiment about organizational fairness because researchers found that long-term stress brings about health problems such as inflammation, which also affected individuals’ perception of fairness (Elovainio et al., 2010).

### Perceived stress, demographic characteristics, and public acceptance

Under different conditions of perceived stress, the differences in public acceptance of WTE incineration facilities caused by demographic characteristics are consistent different. First, although women’s acceptance of potentially hazardous facilities is significantly lower than that of men because of their higher risk perception, the conclusion has been verified (Flynn et al., 1994; Gustafsod, 1998). However, Weiner et al. (2013) concluded in a survey on tolerance of environmental risks. In the general public, men’s tolerance for the environmental risks of potentially hazardous facilities is higher than that of women. However, in communities with high environmental stress, the gender gap in risk tolerance no longer exists, because men and women have the same resistance when facing threats from the environment for a long time. In addition, according to Liu et al. (2018) survey on the public acceptance of WTE projects by residents living near WTE facilities, the results also show that gender differences do not lead to significant differences in public acceptance, because all residents living in WTE incineration plants are subjected to environmental stress from facilities, resulting in consistently low public acceptance.

Secondly, the educational background may change one’s cognitive level to a certain extent, so the educational background is also an important factor affecting the public’s acceptance of potentially hazardous facilities (Choi et al., 2000). Sun and Zhu (2014) found that public with higher education level have higher acceptance of nuclear energy, because they know more about nuclear energy. However, according to Yang et al. (2006), public who also live in communities with high environmental stress (near nuclear power plants) in China shows that, contrary to previous conclusions, the higher education levels, the lower acceptance. This is largely because of that people with higher education are more concerned about their living environment and health, thus overestimating the risk of nuclear power.

Therefore, throughout past research results, perceived stress can influence individuals’ acceptance of potentially hazardous facilities, and this may lead to changes in the influence of demographic characteristics on public acceptance. For the general public, male, with highly education will have higher public acceptance of potentially hazardous facilities. For groups with high perceived stress, the difference in public acceptance caused by individual demographic characteristics will change. However, previous studies mainly focused on local community residents living around potentially hazardous facilities (Greenberg and Schneider, 1995; Weiner et al., 2013), the research on the influence of perceived stress on public’s acceptance of laymen (those who live far away and have nothing to do with the industry) is mostly lacking.

## Research design

### Overall research framework

On the basis of emphasizing the differences in public acceptance caused by demographic characteristics, the study focuses on laypeople and explores whether gender, age, educational attainment, and income still have a significant difference on public acceptance under different perceived stress groups sample.

**Figure 1:**
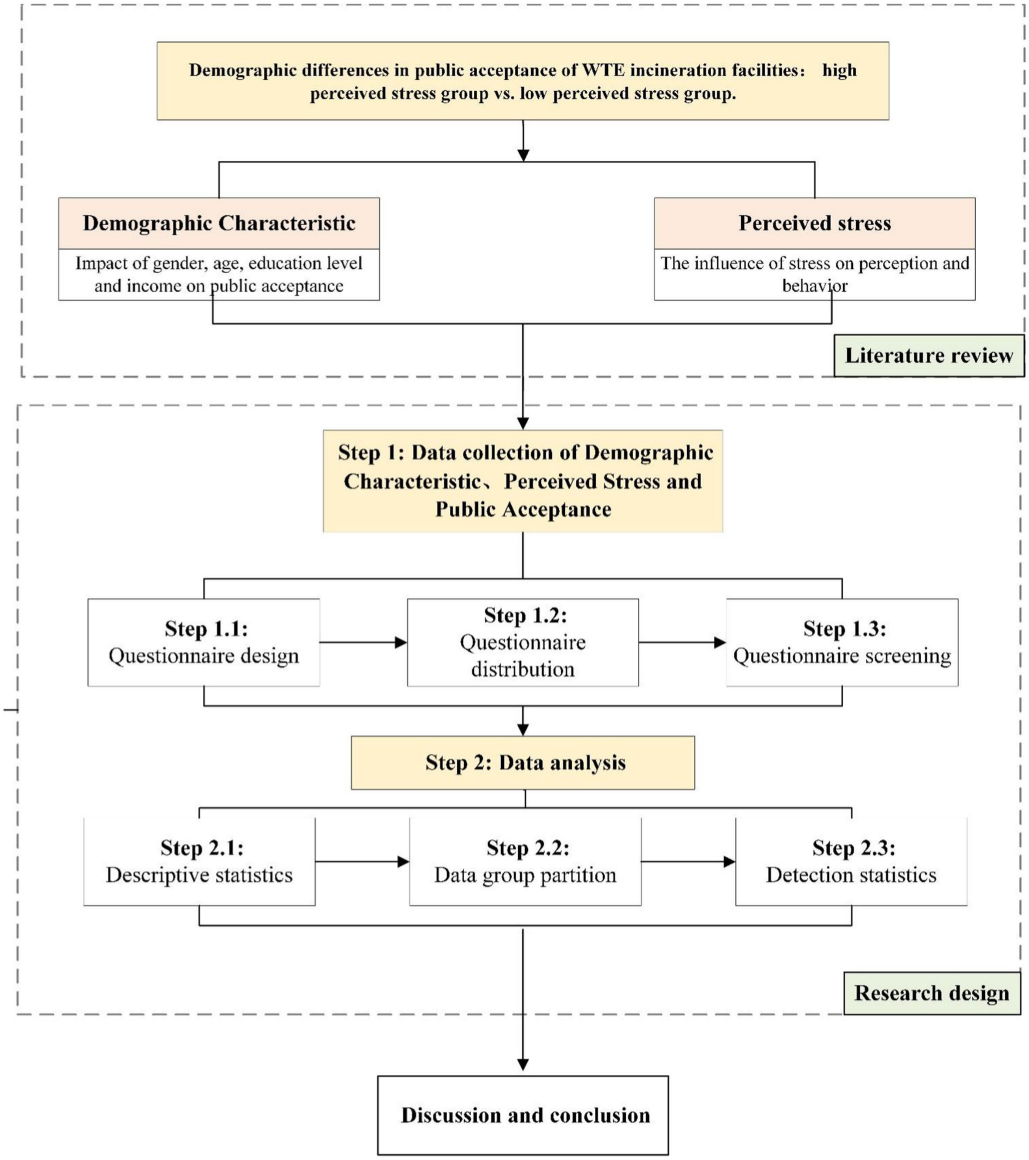
Research framework.

Figure 1 shows the research framework of empirical research in the study. First, according to the literature review, the study designs a questionnaire that included three parts: demographic information, perceived stress level test, and the test of WTE incineration acceptance. Second, a large-scale questionnaire survey was conducted in one city in East China, Central China, and South China. All the collected samples were divided into high perceived stress group samples and low perceived stress group samples according to the scores of the perceived stress level test. Third, T-test and one-way ANOVA were used to test the three groups of samples, respectively, to explore whether the differences in public acceptance of WTE incineration facilities caused by demographic characteristics are consistent in residential groups under different perceived stress.

### Questionnaire design

The research questionnaire consists of three parts. The first part is a survey of demographic information, such as gender, age, and education attainment. According to China’s classification of educational attainment, educational attainment can be divided into two dimensions: Low educated (primary school to high school) and High educated (junior college, undergraduate, postgraduate, and above). According to the standards put forward by the United Nations World Health Organization, the age division can be divided into two dimensions: 18–44 years old (young people), 45 years old, and above (middle-aged and elderly people). The second part is the scale for measuring respondents’ perceived stress. The measurement items are all drawn from the PSS 14 (Perceived Stress Scale 14) proposed by Cohen et al. (1983), which has been tested in the past 30 years, and the results show that it has good reliability and validity and has become one of the most popular stress perception scales in the world. The third part is three measurement items raised by Liu et al. (2018), which related to respondents’ acceptance towards WET incineration projects.

### Samples and data collection

Three representative second-tier cities in China, namely Hangzhou (eastern city), Wuhan (central city), and Chongqing (western city), were selected to conduct the survey. The main reasons are as follows: First, cities in different geographical locations have significant differences in cultural and economic characteristics. The survey selected representative cities in each region to satisfy the completeness of the research. Second, these three cities are the centers of the regional economy, with a large population and developed economy, so the demand for WTE incinerators is increasing rapidly. Furthermore, the local governments of these three cities have suffered strong public opposition in the process of introducing potentially hazardous facilities. Therefore, there is urgent to help the local government to improve public acceptance of WTE incinerators by local residents.

The questionnaire survey was conducted from May 13th to May 25th, 2021, in Hangzhou, June 20th to June 30th, 2021, in Wuhan, and August 10th to 15th, 2021 in Chongqing. Local residents living in the selected survey areas were identified as target respondents. Through stratified simple random sampling, 1,200 questionnaires (including 400 in Hangzhou, 400 in Wuhan, and 400 in Chongqing) were sent out to selected respondents. After eliminating the questionnaire with missing items and multiple options, 1,066 valid usable questionnaires were obtained, comprising 335 from Hangzhou (response rate 83.75%), 369 from Wuhan (response rate 92.25%), and 362 from Chongqing (response rate 90.5%).

Table 1 provides the social demographic information of the respondents. Both the gender and age distributions of the respondents are basically consistent with the results of the seventh census in China, which means that the samples collected are representative. Besides, the cities selected in the survey are all economically developed, which makes them have high-quality educational resources. Thus, the distribution of respondents with high educated reached 67.5%, which was higher than the average education level of Chinese residents.

**Table 1:** Demographic characteristics of the respondents.

Profile	Category	Frequency (%)			
		Hangzhou (Eastern)	Wuhan (Central)	Chongqing (Western)	Overall
Gender	Male	169 (50.4%)	189(51.2%)	192 (53%)	550(51.59%)
	Female	166(49.6%)	180 (48.8%)	170 (47%)	516(48.41%)
Age	18–44	221(18.8%)	232(20.1%)	239(24.6%)	692(21.20%)
	≥ 44	114(8.4%)	137(8.7%)	123(8.8%)	374(8.63%)
Education attainment	High Educated	94(28.1%)	132(35.8%)	120(33.1%)	346(32.5%)
	Low Educated	241(71.9%)	237(64.2%)	242(66.9%)	720(67.5%)
Income Level	≤CNY 4000	77(23%)	108(29.3%)	97(26.8%)	282(26.45%)
	CNY 4000–6,000	78(23.3%)	102(27.6%)	106(29.3%)	286(26.83%)
	CNY 6000–8,000	97(29%)	95(25.7%)	97(26.8%)	289(27.11%)
	≥CNY10000	83(24.8%)	64(17.3%)	62(17.1%)	209(19.61%)

### Data analysis

The analysis of data in the study can be divided into the following two stages. First, descriptive statistics were conducted to evaluate the perceived stress level and public acceptance towards WTE incineration facilities. Second, according to the results of PSS 14 (Perceived Stress Scale 14), people with a total score of 43 or more (with an average score of more than 3 points per question) are considered as people with high perceived stress (Zalaquett and Wood, 1998; Yang and Huang, 2003). Therefore, samples with PSS 14 scores greater than or equal to 43 points are divided into high perceived stress group samples, and samples with PSS 14 scores less than 43 points are divided into low perceived stress group samples. T-test is an appropriate statistical method to detect the differences between two groups of data. For more than two groups of data, ANOVA can be used to detect whether there are differences among three groups (or more) of data (Cui and Churchill, 2003). The purpose of the study is to detect whether the differences in public acceptance caused by demographic differences are consistent among different perceived stress groups. So, the current study adopts T-test and variance as the main methods to explore whether the differences in public acceptance of WTE incineration facilities caused by demographic characteristics are consistent in residential groups under different perceived stress.

## Results

### Descriptive statistics

Table 2 shows the descriptive statistical results of all variables of the valid questionnaire. The kurtosis coefficients and skewness coefficients of all variables are following a multivariate normal distribution. Therefore, this data is suitable for T-test and one-way ANOVA.

**Table 2:** Statistical results of the descriptive variables.

Factor	Indicator	Coefficient	Skewness	Mean	Std. dev
Stress	Q1	−0.473	0.107	2.654	0.976
	Q2	−0.193	0.336	2.562	0.976
	Q3	−0.510	0.127	2.741	0.968
	Q4	−0.243	0.38	2.811	0.892
	Q5	−0.297	0.242	2.828	0.929
	Q6	−0.110	0.497	2.632	0.929
	Q7	−0.242	0.341	2.728	0.931
	Q8	−0.309	0.093	2.720	0.855
	Q9	0.076	0.488	2.659	0.863
	Q10	−0.252	0.253	2.802	0.951
	Q11	−0.236	−0.04	2.801	0.856
	Q12	−0.070	−0.526	3.380	0.981
	Q13	−0.422	0.441	2.710	0.998
	Q14	0.140	0.378	2.617	0.889
Public acceptance	F1	−0.539	−0.235	3.052	1.024
	F2	−0.708	0.063	2.766	1.037
	F3	−0.583	0.203	2.544	1.05

As shown in descriptive statistics the scores of three terms (F1, F2, and F3) used to express the public’s recognition of WTE incineration facilities are 3.05, 2.77, and 2.54 respectively, which shows that the public acceptance of WTE incineration facilities is low.

### Overall sample test

Table 3 shows the results of using T-test and one-way ANOVA to test whether gender, age, education attainment, and income level have a significant difference on public acceptance. In terms of gender, the average score of males for public acceptance terms is 8.829, and that of the female is 7.864. Male’s public recognition of WTE incineration facilities is significantly higher than female’s (*p* = 0.000). In terms of educational attainment, the average score of the public acceptance items of the group with high educational attainment is 8.501, while that of the group with low educational attainment is 8.072 points. Public acceptance of WTE incineration facilities by the group with higher educational attainment has significantly higher than that of the group with low education (*p* = 0.018). However, there is no significant difference in the age and income level on public acceptance.

**Table 3:** Test results of the difference of demographic characteristics on public acceptance (overall sample).

Profile	Category	Mean	Standard deviation	F (for ANOVA)/t (for T-test)	P
Gender (T-test)	Male	8.829	2.689	5.809	0.000 (^***^)
	Female	7.866	2.719		
Education attainment (T-test)	Low educated	8.072	2.961	−2.396	0.017 (^*^)
	High educated	8.501	2.624		
Age (T-test)	18–44	8.279	2.264	−1.305	0.178
	>44	8.516	2.931		
Income (ANOVA)	≤CNY4000	8.486	0.152	0.907	0.437
	CNY4001-CNY6000	8.497	0.161		
	CNY6001-CNY10000	8.197	0.170		
	>CNY10000	8.239	0.197		

***p < 0.001 and *p < 0.05.

In this survey, the overall samples were divided into a high perceived stress group sample (the score of PSS14 was 43–70) and a low perceived stress group sample (the score of PSS14 was 14–42). Among them, there were 283 questionnaires in the high perceived stress group sample (26.55% of the total sample) and 783 questionnaires in the low perceived stress group sample (73.45% of the total sample).

Table 4 explores the difference in public acceptance between high perceived stress group samples and low perceived stress group samples by using a t-test. The average score of the public acceptance terms of the samples in the high perceived stress group is only 5.986, while the average score of the samples in the low perceived stress group is 9.211, with a significant difference (p = 0.000).

**Table 4:** The result of testing the difference in public acceptance among different perceived stress groups.

Profile	Category	Mean	Standard deviation	t	P
Perceived stress	High Perceived stress (N = 283)	5.986	2.471	22.229	0.000 (^***^)
	Low Perceived stress (N = 783)	9.211	1.946		

***p < 0.001.

### Group sample test

#### High perceived stress group sample

Table 5 shows the test results of the T-test and one-way ANOVA in high perceived stress group samples. It can be found that differences in gender, age, educational attainment, and income level have no significant difference on public acceptance.

**Table 5:** Test results of the difference of demographic characteristics on public acceptance. (High perceived stress group samples).

Profile	Category	Mean	Standard deviation	F (for ANOVA)/t (for T-test)	P
Gender	Female(N = 127)	6.189	1.967	1.588	0.113
	Male(N = 156)	5.821	1.919		
Education attainment	Low education (N = 94)	5.809	2.191	−1.082	0.280
	High education(N = 189)	6.074	1.812		
Age	18-44(N = 182)	6.027	1.882	2.377	0.63
	>44(N = 101)	5.911	2.065		
Income	≤CNY4000(N = 79)	6.367	2.027	1.854	0.138
	CNY4001-6000(N = 71)	6.014	1.996		
	CNY6001-10000(N = 91)	5.67	1.777		
	>CNY10000(N = 42)	5.905	1.998		

#### Low perceived stress group sample

Table 6 shows the test results of the T-test and one-way ANOVA in low perceived stress group samples. There are significant differences in public acceptance in terms of gender (p = 0.018); There are significant differences in public acceptance in terms of age (*p* = 0.032), among which older people have higher public acceptance than younger people. Age has no significant difference on public acceptance.

**Table 6:** Test results of the difference of demographic characteristics on public acceptance. (Low perceived stress group samples).

Profile	Category	Mean	Standard deviation	F (for ANOVA)/t (for T-test)	P
Gender	Male(N = 423)	9.622	2.351	1.16	0.000 (^***^)
	Female(N = 360)	8.755	2.531		
Education attainment	Low education(N = 252)	8.917	2.764	−2.234	0.018 (^*^)
	High education (N = 531)	9.365	2.308		
Age	18-44(N = 510)	9.082	2.3904	−2.096	0.032 (^*^)
	>44(N = 273)	9.480	5.5996		
Income	≤CNY4000(N = 203)	9.31	2.231	1.825	0.141
	CNY4001-CNY6000 (N = 215)	9.316	2.4134		
	CNY6001-CNY10000 (N = 198)	9.359	2.5306		
	>CNY10000(N = 167)	8.826	2.7198		

***p < 0.001 and *p < 0.05.

## Discussion and implications

As shown in Table 3, the score of the items used to express public acceptance is only 2.54, 2.77, and 3.05, respectively, which implies that the residents living in second-tier cities in China have a low public acceptance of WTE incineration projects. However, the result is much higher than the score drawn by Liu et al. (2019) and Zhou et al. (2022) using the same questionnaires to investigate local residents near WTE incinerators in Hangzhou and Ningbo, Zhejiang province, and Nanjing, Jiangsu province, with only about 2 points. This result further confirms that residents living near potential hazardous facilities have a more negative attitude towards the facilities (Wang et al., 2019). In addition, Table 4 shows that the public acceptance of the respondents in the low perceived stress group is significantly higher than that in the high perceived stress group (*p* = 0.000), which proves that perceived stress plays an important role in the public acceptance of WTE projects (Greenberg, 2009). Thus, reducing residents’ perceived stress can be a logical and reasonable measure to deal with the strong public opposition to potentially hazardous facilities. In fact, reducing residents’ perceived stress by introducing and improving the local medical system and improving the local social security system has gradually become one of the popular strategies for local governments to improve public acceptance of potentially hazardous facilities (Wang et al., 2014; Saladino et al., 2020; Simpson et al., 2021).

The results of the overall sample test provide strong empirical support for the difference of demographic characteristics on public acceptance. First, males have higher public acceptance than females, which is consistent with previous studies, such as the research results of Wang and Kim (2019) and Kahan et al. (2007). On the one hand, Karpiak and Baril (2008) hold that females are more eco-centric and pay more attention to the environment than males, so females are more sensitive to the environmental risks brought by potentially hazardous facilities. On the other hand, a key reason for gender differences in public acceptance is related to the different roles of males and females in society. As guardians of health and safety in the family, females are more cautious and easier to learn from the disasters and deaths caused by past technical failures. Males are more willing to discuss the risks and economic benefits of facilities from the technical point of view, which leads females to reject potentially hazardous facilities more than males (Freudenburg and Davidson, 2007; Miller, 2012). Second, people with higher education are more likely to accept WTE incineration facilities than people with low education. Educational attainment may cause individual differences in obtaining information and resources, thus affecting people’s views on technological risks, and indirectly affecting the acceptability of technology (Duan, 2010). Moreover, with the improvement of the educational attainment of individuals, people can feel the importance of facilities, that is, the positive benefits brought by potentially hazardous facilities, whether it is the solution of environmental problems or the promotion of the local economy (Choi et al., 2000; Mitchell et al., 2007). Therefore, bridging the gap in residents’ knowledge about potential hazardous facilities can be an important measure to promote public acceptance of WTE incineration facilities (Sun and Zhu, 2014). When the government increases residents’ awareness of potentially hazardous facilities through timely risk communication meetings and hearings, residents can reduce perceived risks and improve perceived benefits (Chung and Kim, 2009; Ferry and Eckersley, 2015).

In particular, the test results from the high perceived stress groups samples do not support the difference of gender on public acceptance as indicated by the overall samples. This phenomenon may be associated with physical stress responses. On the one hand, according to Traczyk et al. (2015), under the state of high perceived stress, both males and females can easily produce the risk images of past serious accidents, which leads to higher risk perception. Because the negative information such as air pollution caused by WTE incineration facilities makes people who feel high stress bring more risk perception, which makes their public acceptance close and low (Kozyreva et al., 2020). Thus, the finding of the high perceived stress group sample is contrary to the result of the overall sample may be first due to higher risk perception caused by perceived stress. On the other hand, studies from the medical field show that when the individual perceives excessive stress, it will lead to negative abnormal physiological reactions and these reactions often lead to psychological abnormalities such as anxiety, tension, depression, fear, decreased personal efficacy and behavioral disorders such as behavior withdrawal and aggressive behavior increase (Cannon, 1939; Lupien et al., 2009). Therefore, in the high perceived stress groups samples in the study, both males and females have similar physiological and psychological reactions when they bear the same high stress, which means males and females may also have a tendency to escape or resist WTE incineration facilities because of the stress reaction, which also reduces the difference of gender on public acceptance (Weiner et al., 2013).

Similarly, the test results of the sample of the high perceived stress group also failed to support the difference of educational attainment on public acceptance of WTE incineration facilities. As emphasized by the cognitive-affective system theory put forward by Mischel and Shoda (1995), when the change of emotion makes the old cognition and the new emotion does not match, it will lead to the change of cognition, thus alleviating the contradiction between cognition and emotion (Schultheiss et al., 2008). Hence, although the cognition brought by excellent educational background can make the public have a certain cognition of potentially hazardous facilities (Stoutenborough et al., 2013), when the negative emotions caused by perceived stress conflict with cognition, people may overturn the limited cognition of potentially hazardous facilities and hold a negative and exclusive attitude towards them. Meanwhile, Skinner and Wellborn (2019) pointed out that under the state of psychological stress, the individual’s response includes not only the intentional response of controlling the danger, acquiring ability and autonomy, but also the individual’s unconscious psychological defense mechanism. In fact, this kind of defense mechanism may make people with higher education want to control potential dangers through their limited knowledge, which makes it easier to overestimate the influence of potentially hazardous facilities, thus leading to resistance (Yang et al., 2006).

In relation to age, no significant difference of age on public acceptance was observed in the overall sample and high perceived stress group sample, but in the low perceived stress group sample, it was reported that older people had higher public acceptance. There may be two reasons for this result. First, young people are faced with family responsibilities of getting married, giving birth and raising children, so they are more worried about the threat posed by WTE incineration facilities to families and young children, and hold a rejection attitude (Huang et al., 2013; Roh and Kim, 2017). Secondly, in the age when middle-aged and elderly people were born, not only did local governments pay insufficient attention to environmental problems but also the way of controlling environmental problems at that time was not effective enough (Zhu et al., 2014; Xie, 2020). Therefore, after going through a worse environment, they can not only realize the pollution problems caused by urban garbage, but also hope to solve these problems through effective solutions, according to that, they can better accept the benefits that WTE incineration facilities bring to their living environment (Greenberg, 2009). However, with the increase in perceived stress, we found that public acceptance of the WTE incineration facilities by the elderly declined obviously, which was the same as that of the young people. The most possible reason for this phenomenon is the different sources of stress. The main reason for the high perceived stress of the elderly is their health problems, which suggests that the elderly with high perceived stress may face more health-related problems than the elderly with low perceived stress (Folkman et al., 1987; Tsai et al., 2015). Therefore, the elderly with high perceived stress may show more demand for health, which will also make the elderly cautious and skeptical about WTE incineration facilities in the current study (Wang et al., 2001).

## Conclusion

In light of the important influence of demographic characteristics on public acceptance of WTE incineration facilities, the current study explores whether demographic characteristics have a significant difference on public acceptance in different groups of perceived stress using a questionnaire and T-test (one-way ANOVA). The research results provide favorable empirical supports that the difference of demographic characteristics on public acceptance produces different results when the perceived stress changes through a questionnaire survey conducted among residents of three second-tier cities in China. The results are as follows:

1. The public acceptance of the WTE incineration project in the low perceived stress group is higher than that in the high perceived stress group.
2. In the overall sample, males have higher public acceptance than females; Groups with high education attainment have higher public acceptance than those with low education attainment.
3. The difference of demographic characteristics (gender, educational attainment, and age) on public acceptance of WTE incineration facilities have been changed in different groups of perceived stress levels.

This conclusion not only enriches the relevant knowledge system of environmental psychology and demographic characteristics research in NIMBY infrastructure projects, but also helps to provide theoretical support for the government, enterprises, and relevant NIMBY professionals to make more effective decisions in the future.

However, the current study still has some limitations. First, the public survey only makes choices for representative second-tier cities in China, which has not been poorly verified in different socio-economic environments. In the future, we will conduct large-scale research on the public with other diverse socio-economic backgrounds (such as first-tier cities with developed economies or poor rural areas) to further verify the influence of perceived stress on the public acceptance of NIMBY infrastructure. Secondly, the study only finds that perceived stress has an impact on public acceptance, Whether the unspecified stress (such as environmental stress, health stress, and employment stress) affects the public acceptance is not divided clearly. Therefore, we will also make a more detailed study on stress to confirm which kind of stress plays a major role in influencing public acceptance. Finally, in different perceived stress environments, the difference of age on public acceptance has not been supported by abundant literature. We will also do relevant research and design in the future to find the potential reasons.

## Data availability statement

The original contributions presented in the study are included in the article/Supplementary material; further inquiries can be directed to the corresponding author.

## Author contributions

JC: writing—original draft, investigation, and formal analysis. XH: writing—original draft and resources. YL: conceptualization, methodology, project administration, and funding acquisition. CC: software, visualization, and data curation. YS: methodology, validation, and supervision. YZ: software, methodology, and validation. All authors contributed to the article and approved the submitted version.

## Funding

This work was supported by the National Natural Science Foundation of China (NSFC; grant nos. 72072165, 71672180, and 72001079), the Soft Science Research Program of Zhejiang Province, China (grant no. 2020C35055), and the Research Foundation of Education Bureau of Zhejiang Province, China (Grant No. Y202045460).

## Conflict of interest

The authors declare that the research was conducted in the absence of any commercial or financial relationships that could be construed as a potential conflict of interest.

## Publisher’s note

All claims expressed in this article are solely those of the authors and do not necessarily represent those of their affiliated organizations, or those of the publisher, the editors and the reviewers. Any product that may be evaluated in this article, or claim that may be made by its manufacturer, is not guaranteed or endorsed by the publisher.
