# Supplementary material for: Demographic differences in public acceptance of waste-to-energy incinerators in China: High perceived stress group vs. low perceived stress group
Source: Front Psychol. 2022 Oct 26;13:948653. doi: 10.3389/fpsyg.2022.948653 (PMC9643326; doi:10.3389/fpsyg.2022.948653)
Supplement: Supplementary file 1 [file Table_1.DOCX]

**Appendix A**

**Part1 Demographic information您的个人信息**

Your age 您的年龄：

□18-44 (18-44岁) □45 and above (45岁及以上)

Your gender 您的性别为：

□Male 男 □Female 女

Your education level 您的受教育程度：

□ Low educated (primary school to high school) 初等教育(小学到高中)

□ High educated(junior college, undergraduate, postgraduate, and above) 高等教育(高中及以上）

Your monthly income level 您的月收入水平：

□CNY4000 and below 4000元及以下 □CNY4001-6000 4001-6000元

□CNY6001-10000 6001-10000元 □CNY10000 and above 10000元以上

**Questionnaire（测量条款）**

**Part2 PSS ( Perceived Stress Scale) 14**

**Notice（注意）**：In the following items（在后续题项中）,

1- Never（从来没有） 2- Almost never（基本没有）

3- Sometimes（偶尔） 4- Fairly often（经常）

5- Very often（频繁）

| No.  编号 | Items  题项 | Options  选项 | | | | |
| --- | --- | --- | --- | --- | --- | --- |
| Q1 | In the last month, how often have you been upset because of something that happened unexpectedly?  在过去的一个月中，您因为意外发生而感到沮丧 | 1 | 2 | 3 | 4 | 5 |
| Q2 | In the last month, how often have you felt that you were unable to control the important things in your life?  在过去的一个月中，您觉得自己无法控制生活中的重要事情？ | 1 | 2 | 3 | 4 | 5 |
| Q3 | In the last month, how often have you felt nervous and "stressed"  在过去的一个月中，您感到紧张和“压力过大” | 1 | 2 | 3 | 4 | 5 |
| Q4 | In the last month, how often have you dealt successfully with irritating life hassles?  在过去的一个月中，您觉得成功地处理了烦人的生活难题 | 1 | 2 | 3 | 4 | 5 |
| Q5 | In the last month, how often have you felt that you were effectively coping with important changes that were occurring in your life?  在过去的一个月中，您觉得自己有效地应对了生活中发生的重要变化 | 1 | 2 | 3 | 4 | 5 |
| Q6 | In the last month, how often have you felt confident about your ability to handle your personal problems?  在过去的一个月中，您觉得自己有信心处理个人问题 | 1 | 2 | 3 | 4 | 5 |
| Q7 | In the last month, how often have you felt that things were going your way?  在过去的一个月中，您感觉事情进展顺利 | 1 | 2 | 3 | 4 | 5 |
| Q8 | In the last month, how often have you found that you could not cope with all the things that you had to do?  在过去的一个月中，您觉得自己难以应付所有必须完成的事 | 1 | 2 | 3 | 4 | 5 |
| Q9 | In the last month, how often have you been able to control irritations in your life?  在过去的一个月中，您觉得自己能够控制生活中的烦恼 | 1 | 2 | 3 | 4 | 5 |
| Q10 | In the last month, how often have you felt that you were on top of things?  在过去的一个月中，您觉得自己处于最佳状态 | 1 | 2 | 3 | 4 | 5 |
| Q11 | In the last month, how often have you been angered because of things that happened that were outside of your control?  在过去的一个月中，您觉得自己因为无法控制的事情而感到生气 | 1 | 2 | 3 | 4 | 5 |
| Q12 | In the last month, how often have you found yourself thinking about things that you have to accomplish?  在过去的一个月中，您觉得自己总在思考自己必须完成的事 | 1 | 2 | 3 | 4 | 5 |
| Q13 | In the last month, how often have you been able to control the way you spend your time?  在过去的一个月中，您觉得自己能够自有支配自己的时间 | 1 | 2 | 3 | 4 | 5 |
| Q14 | In the last month, how often have you felt difficulties were piling up so high that you could not overcome them?  在上个月，您觉得自己所面临的困难堆积如山以至于无法克服 | 1 | 2 | 3 | 4 | 5 |

**Part3**

**Notice（注意）**：In the following items（在后续题项中）,

1- Extremely Disagree（极其不同意） 2- Disagree（不同意）

3- Moderately（既不同意也不反对） 4- Agree（同意）

5- Extremely Disagree（极其同意）

| No.  编号 | Items  题项 | Options  选项 | | | | |
| --- | --- | --- | --- | --- | --- | --- |
| Thinking about the WTE incineration plant located in your community, to what extent do you agree or disagree with the following statements?  若在您所在的地区建设或运营垃圾焚烧发电厂，您觉得： | | | | | | |
| S20 | It is scientific to construct and operate the WTE incineration plant near my community.  在本地建设垃圾焚烧发电厂是科学的. | 1 | 2 | 3 | 4 | 5 |
| S21 | I would be willing to accept the WTE incineration plant being constructed /operated near my community.  会支持在本地区附近建设垃圾焚烧发电厂. | 1 | 2 | 3 | 4 | 5 |
| S22 | I would be willing to try to persuade my relatives and friends to accept the WTE incineration plant being constructed/operated near my community.  会说服亲戚朋友支持在本地区建设垃圾焚烧发电厂. | 1 | 2 | 3 | 4 | 5 |
